# Changes in Volume of Subregions Within Basal Ganglia in Obsessive–Compulsive Disorder: A Study With Atlas-Based and VBM Methods

**DOI:** 10.3389/fnins.2022.890616

**Published:** 2022-06-20

**Authors:** Jiaxiang Chen, Chong Tian, Qun Zhang, Hui Xiang, Rongpin Wang, Xiaofei Hu, Xianchun Zeng

**Affiliations:** ^1^School of Medicine, Guizhou University, Guiyang, China; ^2^Department of Medical Imaging, Guizhou Provincial People's Hospital, Guiyang, China; ^3^Department of Psychology, Guizhou Provincial People's Hospital, Guiyang, China; ^4^Department of Radiology, Southwest Hospital, Third Military Medical University (Army Medical University), Chongqing, China

**Keywords:** obsessive-compulsive disorder, basal ganglia, voxel-based morphometry, Freesurfer, statistical parametric mapping, Atlas-based analysis

## Abstract

**Background:**

The role of basal ganglia in the pathogenesis of obsessive–compulsive disorder (OCD) remains unclear. The studies on volume changes of basal ganglia in OCD commonly use the VBM method; however, the Atlas-based method used in such research has not been reported. Atlas-based method has a lower false positive rate compared with VBM method, thus having advantages partly.

**Objectives:**

The current study aimed to detect the volume changes of subregions within basal ganglia in OCD using Atlas-based method to further delineate the precise neural circuitry of OCD. What is more, we explored the influence of software used in Atlas-based method on the volumetric analysis of basal ganglia and compared the results of Atlas-based method and regularly used VBM method.

**Methods:**

We analyzed the brain structure images of 37 patients with OCD and 41 healthy controls (HCs) using the VBM method, Atlas-based method based on SPM software, or Freesurfer software to find the areas with significant volumetric variation between the two groups, and calculated the effects size of these areas.

**Results:**

VBM analysis revealed a significantly increased volume of bilateral lenticular nucleus in patients compared to HCs. In contrast, Atlas-based method based on Freesurfer revealed significantly increased volume of left globus pallidus in patients, and the largest effect size of volumetric variation was revealed by Freesurfer analysis.

**Conclusions:**

This study showed that the volume of bilateral lenticular nucleus significantly increased in patients compared to HCs, especially left globus pallidus, which was in accordance with the previous findings. In addition, Freesurfer is better than SPM and a good choice for Atlas-based volumetric analysis of basal ganglia.

## Introduction

Obsessive–compulsive disorder (OCD) is a common mental illness with a prevalence of about 1–3% (Fontenelle and Hasler, 2008). The symptoms include obsession and compulsion. Obsession is intrusive and repetitive thoughts, images, impulses, or demands of the patients; compulsion is repetitive behaviors or mental activities to subside the discomfort caused by obsession or bring a sense of “completeness” (Mataix-Cols et al., 2005; Bloch et al., 2008). For example, concerns about stains can lead to repeated cleaning. Most patients realize the irrationality of these symptoms and take the initiative to control them, but they usually fail. OCD commonly co-occurs with anxiety and depression, which add to psychological and physical discomfort and decrease quality of life, and has been listed as one of the top 10 leading causes of disability worldwide by World Health Organization (WHO) (Lopez and Murray, 1998).

Basal ganglia plays a key role in the pathogenesis of OCD. This subcortical structure is composed of the caudate, lenticular nucleus (putamen and globus pallidus), nucleus accumbens, and other areas. At the molecular level, neurotransmitters such as 5-HT, dopamine, and glutamate in the striatum of basal ganglia are dysregulated in OCD. For example, the expression of dopamine D2 receptor decreased in the striatum (Nikolaus et al., 2010; Olver et al., 2010), and the knockout of glutamatergic gene SAPAP3 mainly expressed in striatum will lead to compulsive grooming behavior of mice (Burguière et al., 2013). Damage to the basal ganglia induced by antibodies can also lead to OCD (Endres et al., 2022). At the neural circuits level, the abnormality of basal ganglia activity leads to cognitive-affective dysfunction, which is manifested as the patients' negative interpretations of obsession that can result in compulsion (Schwartz, 1998). In addition, the increased activation of caudate in patients with OCD is related to excessive habits (Gillan et al., 2016), and the decreased activity of globus pallidus and posterior caudate leads to deficient goal-directed control (Rasgon et al., 2017), resulting in a rigid way. As such, the abnormality of basal ganglia is one of the main pathogenesis of OCD. However, the results of these OCD brain studies had large heterogeneity. For example, a VBM-based meta-analysis found an increased volume of lenticular nucleus in patients with OCD (Radua and Mataix-Cols, 2009), while another VBM-based multicenter study did not reveal similar results (De Wit et al., 2014). Therefore, further studies are still needed to clarify the role of basal ganglia in the pathogenesis of OCD.

Compared with brain function analysis, volume analysis has lower sensitivity to external disturbance and better repeatability, and so widely applied in the studies of neuropsychiatric diseases. VBM analysis is the most commonly used method in the study of basal ganglia volume in OCD. For example, Tang et al. (2016) used the VBM method to analyze the volume of cortico–striato–thalamo–cortical (CSTC) circuits in OCD, which revealed decreased volume of gray matter in bilateral striatum of patients. Another study found no significant difference in basal ganglia volume between refractory OCD and healthy people using the VBM method (Tang et al., 2013). However, this method has a high false-positive rate due to one single voxel as minimum analysis unit. By contrast, Atlas-based method parcellates the individual brain into several areas according to the anatomical atlas of the brain, then calculates their volumes, and carries out subsequent statistical analysis with each area as minimum analysis units. Compared with the VBM method, Atlas-based method has a lower false-positive rate caused by noise (Lieberman and Cunningham, 2009) and fewer comparisons, which reduce the strictness of multiple comparison correction, thus having advantages partly. However, Atlas-based method used to analyze basal ganglia volume in OCD has not been reported.

To further delineate the precise neural circuitry of OCD, we analyzed the volume of subregions within the basal ganglia by Atlas-based method using SPM and Freesurfer software. In addition, we also compare the results of Atlas-based method and regularly used VBM method to understand the differences between the two methods.

## Materials and Methods

### Participants

A total of 41 patients who met DSM-V criteria for OCD were collected from the Department of psychology in Guizhou Provincial People's Hospital from December 2020 to December 2021. These patients also met: (1) age of 18–65 years; (2) had not been treated systematically for OCD (Boedhoe et al., 2018; Bruin et al., 2020; Kong et al., 2020); (3) right-handedness (Jang et al., 2017); (4) no psychiatric comorbidities (except mild or moderate depression; Honda et al., 2017; Naaijen et al., 2018); (5) no history of nervous system diseases (Honda et al., 2017; Naaijen et al., 2018); (6) no history of intracranial injury (Honda et al., 2017); (7) no substance abuse and dependence in recent 1 year (Honda et al., 2017); (8) no neurodevelopmental delay (Naaijen et al., 2018); (9) no contraindications for participating in MRI.

A total of 41 healthy controls (HCs) matched to the patients in age and sex were recruited through the Internet. The Chinese version of MINI-International Neuropsychiatric Interview (MINI) conducted by two psychologists with the title of attending physician showed no mental illness in these healthy controls. In addition, the HCs also meet: (1) right-handedness; (2) no history of nervous system diseases; (3) no history of intracranial injury; (4) no substance abuse and dependence in recent 1 year; (5) no neurodevelopmental delay; (6) no contraindications for participating in MRI.

All subjects signed informed consent. This study was approved by the Institutional Review Board of Guizhou Provincial People's Hospital and in accordance with the Declaration of Helsinki.

### Image Acquisition

Prior to the MR scan, all subjects were asked to remove personal metal products and keep their heads motionless during the scan. High-resolution whole-brain 3D T1 structural images (TR/TE = 8.5/3.2 ms, TI = 450 ms, FA = 15°, FOV = 256 mm × 256 mm, matrix = 256 × 256, thickness = 1 mm, resolution = 1 mm × 1 mm × 1 mm, 176 axial slices, scan time = 273 s, accelerated by ARC parallel acquisition technology with acceleration factor R = 2) were collected at 3.0T MR scanner (Discovery MR 750w, GE Healthcare, Milwaukee, WI) with a 24-channel head/neck coil. The subjects with obvious motion artifacts and metal artifacts were excluded. In addition, brain MR plain scan (axial T2, sagittal T2, and axial T2flair) was performed to exclude the subjects with organic lesions in the brain.

### VBM: Image Preprocessing

The structural images were segmented, modulated, and normalized following the standard procedure (Farokhian et al., 2017) using Cat12 implemented in Statistical Parametrical Mapping 12 (SPM12) (https://www.fil.ion.ucl.ac.uk/spm/software/spm12/) to generate gray matter, white matter, and cerebrospinal fluid images. Then, the subjects with poor segmentation quality were excluded according to the segmentation reports generated by Cat12. Finally, the qualified gray matter images were smoothed using 4 mm full-width-half-maximum (FWHM).

### Atlas-Based Method

#### Statistical Parametrical Mapping

Gray matter images segmented, modulated, and normalized by Cat12 were smoothed using a 4-mm FWHM. With the 12 subregions within basal ganglia of Brainnetome Atlas (BN atlas) as ROIs, the volume of smoothed gray matter corresponding to each ROI was read and calculated using SPM12 (Suppa et al., 2015; Reading and calculation scripts are provided in Supplementary Materials).

#### Freesurfer

Freesurfer (https://surfer.nmr.mgh.harvard.edu/) is an automated brain parcellation software and proved no significant difference in accuracy from manual tracing (Fischl et al., 2002). In the current study, Freesurfer version 7.1.1 was used to parcellate individual basal ganglia into 12 subregions according to the BN atlas (https://atlas.brainnetome.org/) and then calculate the volume of each subregion in each subject. The parcellation was visually inspected on a visualization software Freeview to exclude the subjects with parcellation errors.

### Statistical Analyses

The normality test and homogeneity of variance test performed in SPSS version 26 showed that neither the age of patients nor HCs were distributed normally, but there was no significant difference in variance between the two groups. Therefore, we conducted a non-parametric test and Chi-square test to test for differences in age and gender between the two groups. The difference was considered as significant if *p* was <0.05.

Statistical analysis of smoothed gray matter was performed in SPM12. A mask was created from BN atlas to limit the statistical analysis steps to basal ganglia. Subsequently, we conducted a two-sample *t*-test on the gray matter of patients and HCs with total supratentorial volume and age as covariates. The results were corrected for multiple comparisons by AlphaSim method (Steward et al., 2019) (FWHM = 4 mm, rmm = 5.00, individual voxel threshold probability = 0.05, number of Monter Carlo simulation = 1,000) to achieve a corrected *p* < 0.05 by a combination of a *p* < 0.05, with a minimum cluster size of 185 voxels.

We conducted the analysis of covariance on the volume of subregions produced by SPM and Freesurfer, respectively, with total supratentorial volume and age as covariates. The results were corrected for Bonferroni multiple comparisons. The difference in volume between the two groups was considered as significant only if corrected *p*-value was <0.05.

The volume values of the areas with volumetric variation revealed by VBM were read and calculated using “spm_ read_ vols” and “spm_ vol” commands. Then we calculated estimated marginal means and standard deviations of volume within all areas with volumetric variation revealed by each method in this study, respectively, with total supratentorial volume and age as covariates. Finally, effect-size estimates were calculated using Cohen's *d* to quantify volume differences between groups, so as to evaluate the ability of the three methods (Atlas-based analysis based on SPM, Atlas-based analysis based on Freesurfer, and VBM analysis) to distinguish between patients and HCs, and was regarded as an indicator of the quality of different analyses.

## Results

There are two patients having organic lesions in the brain (one of left hippocampal cyst and one of lateral ventricle broadening), one patient with obvious motion artifacts and one patient with severe parcellation errors by Freesurfer. Therefore, a total of 37 patients and 41 HCs were included in the follow-up analysis. Table 1 illustrated the clinical and demographic characteristics of the subjects included in the analysis, which indicated no significant difference in age and gender between the two groups.

**Table 1:** Clinical and demographic characteristics of the subjects.

	**OCD patients (*n* = 37)**	**HCs (*n* = 41)**	***p*** **-value**
Age [years, median (IQR)]^a^	25 (7)	25 (11)	0.619
Gender (male/female)^b^	21/16	25/16	0.705
Comorbidities	3 moderate depression/34 none		

a *t-test*.

b *Chi square test*.

**Figure 1:**
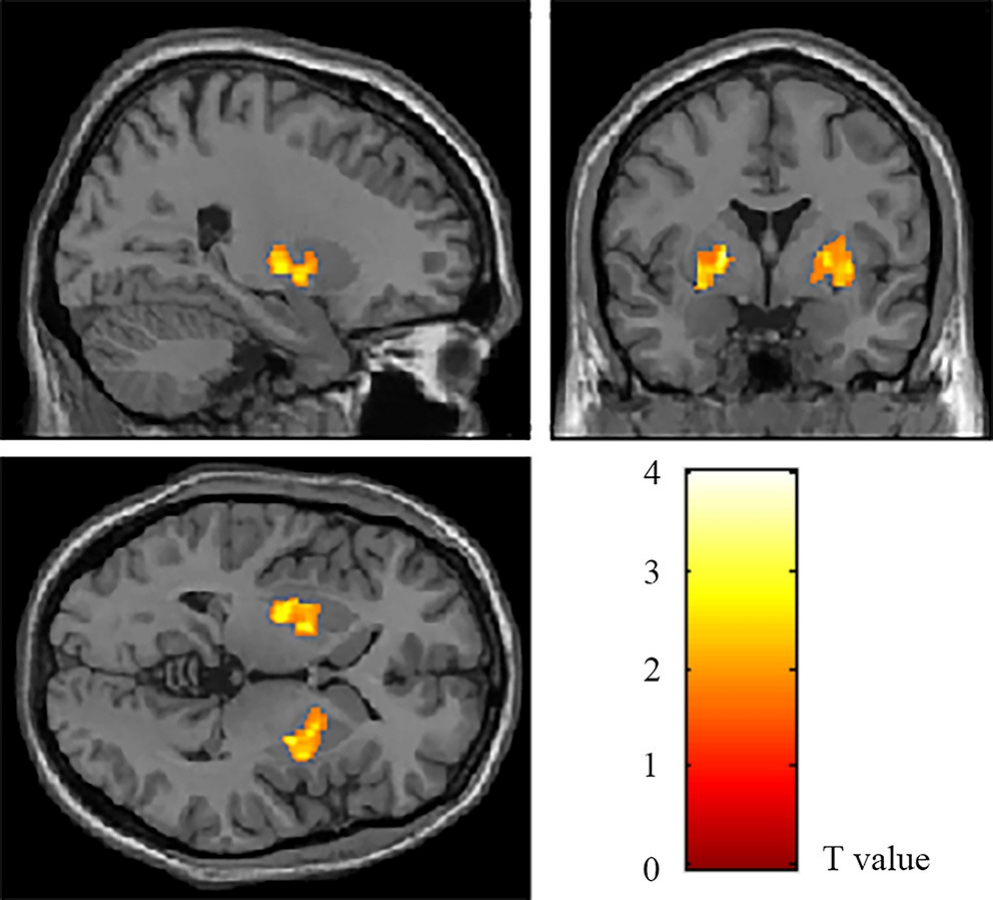
The areas with a significant volumetric difference revealed by VBM analysis. The volume of bilateral lenticular nucleus (ROI_L_ and ROI_R_) increased in OCD patients compared to HCs (*p* < 0.05, AlphaSim corrected).

The VBM analysis revealed a significantly increased volume of gray matter of bilateral lenticular nucleus in the patient's group compared to the HCs group, especially globus pallidus (Table 2, Figure 1). The volume values of the two areas (ROI_L_ and ROI_R_) were read and calculated using “spm_ read_ vols” and “spm_ vol” commands. The subsequent analysis of covariance also showed significant differences in the volume value within ROI_L_ and ROI_R_ between the two groups (Bonferroni-corrected *p*ROI_L_ = 0.023, Bonferroni-corrected *p*ROI_R_ = 0.028).

**Table 2:** The volume of bilateral lenticular nucleus increased in OCD patients compared to HCs.

**Brain areas**	**Side**	**AAL**	**MNI coordinate**			**Cluster size**	***T*** **-value**
			**x**	**y**	**z**		
Lenticular nucleus	L	Globus pallidus	−19.5	−1.5	6.0	734	3.4348
Lenticular nucleus	R	Globus pallidus	24.0	−7.5	4.5	935	3.5012

*x, y, z are the coordinate of T-value peak voxel in MNI space; AAL, Automated anatomical labeling atlas; MNI, Montreal Neurological Institute*.

**Figure 2:**
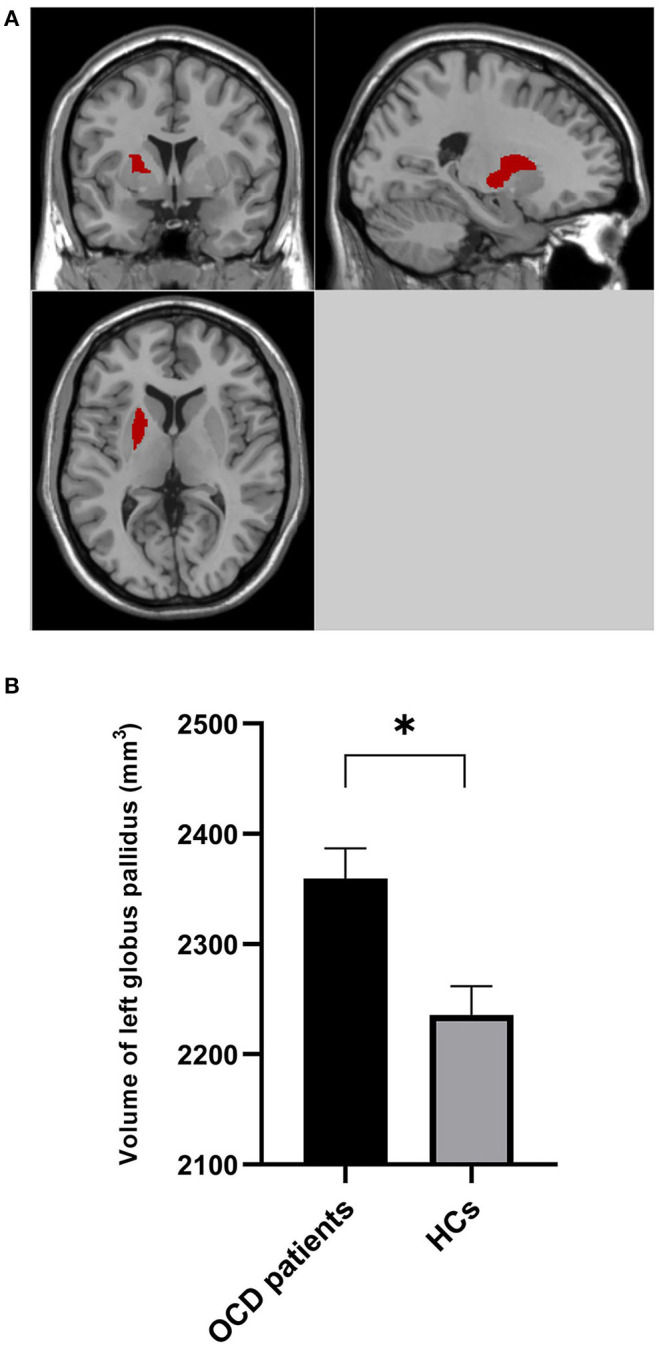
Volume of left globus pallidus was bigger in OCD patients than HCs. **(A)** Left globus pallidus is shown in red. **(B)** The volume of left globus pallidus in OCD patients and HCs controlling for age and total supratentorial volume. ^*^0.01 < *p* < 0.05.

Atlas-based analysis based on SPM revealed increased volume at the trend level of right globus pallidus (5.26%, uncorrected *p* = 0.035), left dorsolateral putamen (4.58%, uncorrected *p* = 0.043), and right dorsolateral putamen (5.55%, uncorrected *p* = 0.047) in patients compared to HCs; however, neither of the three areas survived Bonferroni multiple comparison correction, indicating no significant volumetric difference in subregions within basal ganglia between the two groups (Table 3). However, Atlas-based analysis based on Freesurfer showed a significantly increased volume of left globus pallidus in the patients group compared to the HCs group (5.53%, Bonferroni-corrected *p* = 0.021) (Table 4, Figure 2). In contrast, no other brain areas with significant volume variation or trends were revealed by Freesurfer (uncorrected *p* > 0.05) (Table 4).

**Table 3:** Changes in sub-regional volume of basal ganglia in OCD patients derived from Atlas-based analysis using SPM.

**Subregions of basal ganglia**	***N*** **(patients/HCs)**^**a**^	**uncorrected *p*** ^**b**^	**Bonferroni corrected *p***	**Deviation (%)**
vCa_l	37/41	0.976	1.000	0.05%
vCa_r	37/41	0.510	1.000	1.44%
GP_l	37/41	0.068	0.822	4.51%
GP_r	37/41	0.031	0.377	6.15%
NAC_l	37/41	0.634	1.000	0.66%
NAC_r	37/41	0.586	1.000	−0.77%
vmPu_l	37/41	0.179	1.000	2.73%
vmPu_r	37/41	0.062	0.746	4.20%
dCa_l	37/41	0.882	1.000	−0.32%
dCa_r	37/41	0.598	1.000	−1.10%
dlPu_l	37/41	0.030	0.364	5.67%
dlPu_r	37/41	0.047	0.567	5.55%

a *N is the number of paired patients and HCs*.

b *Analysis of covariance with age and total supratentorial volume as covariates*.

*vCa_l, left ventral caudate; vCa_r, right ventral caudate; GP_l, left globus pallidus; GP_r, right globus pallidus; NAC_l, left nucleus accumbens; NAC_r, right nucleus accumbens; vmPu_l, left ventromedial putamen; vmPu_r, right ventromedial putamen; dCa_l, left dorsal caudate; dCa_r, right dorsal caudate; dlPu_l, left dorsolateral putamen; dlPu_r, right dorsolateral putamen*.

**Table 4:** Changes in sub-regional volume of basal ganglia in OCD patients derived from Atlas-based analysis using Freesurfer.

**Subregions of basal ganglia**	***N*** **(patients/HCs)**^**a**^	**uncorrected *p*** ^**b**^	**Bonferroni corrected *p***	**Deviation (%)**
vCa_l	37/41	0.407	1.000	−1.97%
vCa_r	37/41	0.875	1.000	0.37%
GP_l	37/41	0.002	0.021	5.53%
GP_r	37/41	0.092	1.000	2.93%
NAC_l	37/41	0.815	1.000	−0.47%
NAC_r	37/41	0.931	1.000	−0.18%
vmPu_l	37/41	0.120	1.000	3.19%
vmPu_r	37/41	0.212	1.000	4.32%
dCa_l	37/41	0.651	1.000	1.16%
dCa_r	37/41	0.513	1.000	−2.26%
dlPu_l	37/41	0.667	1.000	0.82%
dlPu_r	37/41	0.665	1.000	0.73%

a *N is the number of paired patients and HCs*.

b *Analysis of covariance with age and total supratentorial volume as covariates*.

*vCa_l, left ventral caudate; vCa_r, right ventral caudate; GP_l, left globus pallidus; GP_r, right globus pallidus; NAC_l, left nucleus accumbens; NAC_r, right nucleus accumbens; vmPu_l, left ventromedial putamen; vmPu_r, right ventromedial putamen; dCa_l, left dorsal caudate; dCa_r, right dorsal caudate; dlPu_l, left dorsolateral putamen; dlPu_r, right dorsolateral putamen*.

Only VBM analysis and Freesurfer analysis revealed areas with significant volumetric variation (ROI_L_, ROI_R_, GP_L). Therefore, we calculated the effect sizes of volume within these areas. As presented in Table 5, the effect size of left globus pallidus revealed by Freesurfer is greater than that of ROI_L_ and ROI_R_ within the bilateral lenticular nucleus revealed by VBM.

**Table 5:** Effect size of regions with significant volumetric variation.

**Methods**	**Region of interest**	**OCD patients**^**a**^ **(volume, cm**^**3**^**)**		**HCs**^**a**^ **(volume, cm**^**3**^**)**		**Effect size**	**Uncorrected *p***	**Bonferroni corrected *p***
		**Mean**	**SD**	**Mean**	**SD**			
VBM analysis	ROI_L_	0.63	0.01	0.59	0.01	3.705	0.011	0.023
	ROI_R_	0.65	0.01	0.61	0.01	3.566	0.014	0.028
Freesurfer analysis	GP_L	2.36	0.03	2.24	0.03	4.622	0.002	0.021

a *Estimated marginal mean and standard deviation (SD) of regional volume with age and total supratentorial volume as covariates*.

*GP_L, left globus pallidus*.

## Discussion

Since there is still no study on the volume of subregions within basal ganglia in OCD with Atlas-based method, we aim to further analyze the morphological changes of basal ganglia in patients with OCD using Atlas-based method to further delineate the precise neural circuitry of OCD, and the feasibility of Atlas-based method was also discussed. The results showed that (1) volume of bilateral lenticular nucleus increased in patients compared to HCs, especially left globus pallidus, which was in accordance with previous findings (Pujol et al., 2004; Radua and Mataix-Cols, 2009; Radua et al., 2010; Boedhoe et al., 2017), (2) the largest variation of volume was revealed by Freesurfer (effect size = 4.622). This study further confirmed the morphological changes of basal ganglia in OCD found in previous studies. In addition, Freesurfer is better than SPM and a good choice for Atlas-based volumetric analysis of basal ganglia.

This study had strict inclusion criteria for the subjects. Firstly, age was limited to 18–65 years to reduce the interference of neurodevelopment or neurodegeneration; secondly, the patients included in this study had not been treated systematically for OCD since the morphology of the brain in OCD could be significantly affected by treatment, which was found in many studies (Boedhoe et al., 2018; Bruin et al., 2020; Kong et al., 2020). Thirdly, there were significant morphological differences mainly in basal ganglia between the people with different handedness (Jang et al., 2017), therefore, only subjects with right-handedness were included in this study. Fourthly, the patients in this study had no other psychiatric comorbidities that will interfere with the results. However, OCD commonly co-occurs with depression. To ensure sufficient sample capacity, patients with mild or moderate depression were also included, and fifthly, the patients with other characteristics influencing the morphology of the brain and mental state, such as nervous system diseases, were excluded.

In the Atlas-based analysis, basal ganglia was parcellated according to the BN atlas. BN atlas was constructed based on the structural and functional connectivity information of 40 healthy adults, and not only confirms some structural differentiations in cytoarchitectonic atlas, but also reveals numerous anatomical subdivisions that have not been found previously (Fan et al., 2016), and is more fine-grained and representative than regularly used AAL atlas generated from only a young man (Tzourio-Mazoyer et al., 2002). Long et al. found that the abnormal volume features determined by the BN atlas instead of the AAL atlas had higher accuracy in distinguishing mild cognitive impairment (MCI) from healthy people (Long et al., 2018), indicating the strength of the BN atlas over AAL atlas. Since there is no cerebellum included in the BN atlas, total supratentorial volume rather than total intracranial volume (tICV) was considered as the covariate in all statistical analyses of the current study.

The previous morphological studies of OCD brain found that different symptom dimensions were related to changes in different brain areas (Van Den Heuvel et al., 2009), but putamen and globus pallidus (lenticular nucleus), whose increase in volume has been reported by many studies, indicate that lenticular nucleus is specific for OCD (Pujol et al., 2004; Radua and Mataix-Cols, 2009; Radua et al., 2010; Boedhoe et al., 2017), and the findings of the current study further confirm this. At present, the abnormality of cortico–striato–thalamo–cortical (CSTC) circuit is the most acceptable neurobiological hypothesis about the pathogenesis of OCD (Brooks and Stein, 2015). As part of the striatum, putamen and caudate receive inputs from the cerebral cortex and transfer the afferents to thalamus *via* globus pallidus, and the thalamus in turn sends the inputs to the cortex directly, forming the CSTC loop (Fazl and Fleisher, 2018). Therefore, as important parts of CSTC circuit, putamen and globus pallidus play a key role in the pathogenesis of OCD. At the transcriptional level, there was differential expression of many OCD candidate genes within putamen of OCD (Lisboa et al., 2019), which might be the basic reason for the abnormality of putamen. At the neurobiological level, task-state functional magnetic resonance (fMRI) studies showed that the activity of putamen in OCD increased during emotional processing (affective paradigm; Rasgon et al., 2017; Thorsen et al., 2018) and decreased while performing tasks relying on executive function (Del Casale et al., 2016), and the activity of globus pallidus involved in goal-directed control decreased on the cognitive paradigm (Rasgon et al., 2017). Therefore, the abnormalities of lenticular nucleus may increase affective processing and impair cognition of patients with OCD, which is considered as cognitive-affective dysfunction. It can lead to patients' overestimation of harm and excessive control over their own thoughts, which further lead to obsession (Obsessive Compulsive Cognitions Working Group, 1997). If the patients cannot adapt to the obsession or control their response to concerns (impaired executive function), compulsion appears (Rachman, 1971; Salkovskis, 1985; Stein et al., 2000). What's more, these abnormalities also lead to excessive habits and deficient goal-directed control in OCD (Gillan et al., 2016). However, there are no other studies using Atlas-based method to analyze basal ganglia volume in OCD. Therefore, more Atlas-based studies and more data sets are needed to verify the repeatability of the current study and further clarify the role of lenticular nucleus in the pathogenesis of OCD.

Since different software will generate discrepant results due to different segmentation algorithms (Mikhael and Pernet, 2019), Freesurfer and SPM were used, respectively, to perform Atlas-based analysis in this study, so as to preliminarily understand the influence of software used in Atlas-based method on the volumetric analysis of basal ganglia. Freesurfer is an automatic processing software with a process including transforming original images to Tailarach space, correction for intensity inhomogeneities, non-brain tissues removal, high dimensional non-linear volumetric alignment to the atlas space, segmentation based on probabilistic atlas and subject-specific measured values, ROI labeling, and subsequently extracting the size of ROI as the volume of corresponding area (Fischl et al., 2004; Desikan et al., 2006). In contrast, SPM directly overlays the ROI of anatomical atlas (mask) on normalized gray matter images and calculates the sum of the volume information in all voxels of this mask as the ROI volume value. In this study, the brain area revealed by Freesurfer survived Bonferroni multiple comparison correction, while these of SPM did not, indicating that Freesurfer is more sensitive to brain morphological changes and has advantages in basal ganglia volume analysis.

In addition, we also performed VBM analysis in our study to understand the heterogeneities and overlaps of the areas with volumetric variation revealed by Atlas-based method and the regularly used VBM method separately. Atlas-based method and VBM method have different strategies for analyzing brain volume. Specifically, VBM conducts a significance test of the difference of each voxel in normalized gray matter so as to generate voxel clusters with a significant volumetric difference as regions of interest (ROIs). The minimum analysis unit of a single voxel makes VBM method sensitive to not only small morphological variations but also local noises that can result in false-positive results. By contrast, Atlas-based method parcellates the brain into several areas according to anatomical atlas, then conducts a significance test of difference for each area instead of each voxel so as to find the areas with significant volumetric variation as ROIs. Compared with the VBM method, Atlas-based method has a lower false-positive rate, but lower sensitivity for small morphological variations (Van Schuerbeek et al., 2016). In the current study, the effect size order was: Atlas-based method based on Freesurfer > VBM method > Atlas-based method based on SPM, with Bonferroni (strong), AlphaSim (weak), and none as corresponding multiple comparison corrections, respectively. Therefore, it is difficult to see which VBM method or Atlas-based method is more advantageous, and previous studies have not revealed which method was better either (Goto et al., 2011a,b; Van Schuerbeek et al., 2016). The heterogeneity in different results seems to be more related to the software used. In our study, Atlas-based analysis was performed by Freesurfer or SPM software, and the VBM analysis was performed by SPM. The difference between the algorithms of these two software may explain why the area with significant volumetric variations revealed by Freesurfer was unilateral, while the areas revealed by the Atlas-based method and VBM method based on SPM were bilateral. For the heterogeneity between the results of VBM method and the Atlas-based method based on SPM, the negative result revealed by the latter may be due to the use of stronger multiple comparison correction than that of the former (Bonferroni vs. AlphaSim), and the former may generate more false positive voxels.

In short, the contrastive analysis discussed above found that heterogeneity of basal ganglia volume analysis results was related to both analysis methods and software, among which software had greater influence. In this study, the brain area with volumetric variation revealed by Freesurfer had the largest effect size (Cohen's d = 4.622) and survived the strongest multiple comparison correction (Bonferroni corrected *p* < 0.05), indicating better ability of Freesurfer to distinguish between patients and healthy people. Therefore, this software is a good choice for Atlas-based method. However, this study cannot find out which of the Atlas-based method and VBM method was more advantageous in basal ganglia volume analysis.

There are some limitations in this study. Firstly, the sample capacity was modest. Secondly, manual tracing was not performed in this study as the gold standard to estimate the real deviation of these methods and software. Thirdly, VBM method in our study only survived relatively weak AlphaSim multiple comparison correction, and the study needs to be further carried out on other datasets. This study preliminary detected volumetric variation within basal ganglia of OCD using the Atlas-based method and further studies are still needed to clarify (1) precise volumetric variation of basal ganglia in OCD and (2) the differences between Atlas-based method and VBM method.

In conclusion, we revealed an increased volume of lenticular nucleus in patients with OCD, especially left globus pallidus, which was in accordance with the previous studies. In addition, the results of basal ganglia volume analysis varied in different analysis methods and software, among which software had greater influence, and Freesurfer is better than SPM and a good choice for Atlas-based volumetric analysis of basal ganglia.

## Data Availability Statement

The original contributions presented in the study are included in the article/Supplementary Materials, further inquiries can be directed to the corresponding author/s.

## Ethics Statement

The studies involving human participants were reviewed and approved by Institutional Review Board of Guizhou Provincial People's Hospital. The patients/participants provided their written informed consent to participate in this study.

## Author Contributions

JC and CT contributed to study concepts and design, data analysis and interpretation, and manuscript drafting. QZ and HX contributed to subjects collection. RW contributed to study concepts and design. XH and XZ contributed to manuscript revision and gave approval of the final version of the submitted manuscript. All authors contributed to the article and approved the submitted version.

## Funding

The study was supported by the Science and Technology Foundation of Guizhou Province (QKHPTRC[2019]5803) and (QKHPTRC[2017]5724).

## Conflict of Interest

The authors declare that the research was conducted in the absence of any commercial or financial relationships that could be construed as a potential conflict of interest.

## Publisher's Note

All claims expressed in this article are solely those of the authors and do not necessarily represent those of their affiliated organizations, or those of the publisher, the editors and the reviewers. Any product that may be evaluated in this article, or claim that may be made by its manufacturer, is not guaranteed or endorsed by the publisher.
