# Supplementary material for: Changes in Volume of Subregions Within Basal Ganglia in Obsessive–Compulsive Disorder: A Study With Atlas-Based and VBM Methods
Source: Front Neurosci. 2022 Jun 20;16:890616. doi: 10.3389/fnins.2022.890616 (PMC9251343; doi:10.3389/fnins.2022.890616)
Supplement: Supplementary file 1 [file Data_Sheet_1.docx]

**Supplementary Materials**

**Matlab script of volume extraction based on SPM**

excel=zeros(78,12);

atlas=spm_read_vols(spm_vol('C:\Users\Administrator\Desktop\code1\rbasalganglia.nii'));

GNo=dir('C:\Users\Administrator\Desktop\code1\sGM');

VolNo=dir('C:\Users\Administrator\Desktop\code1\totalGMvolume');

for num=3:80;

c=spm_read_vols(spm_vol(fullfile('C:\Users\Administrator\Desktop\code1\sGM',GNo(num).name)));

total=sum(sum(sum(c)));

load(fullfile('C:\Users\Administrator\Desktop\code1\totalGMvolume',VolNo(num).name));

totalvol=S.subjectmeasures.vol_abs_CGW(2);

for atlasno=219:230;

volume=sum(sum(sum(c(find(atlas==atlasno)))))*totalvol/total;

excel(num-2,atlasno-218)=volume;

end

clear S;

end

xlswrite('C:\Users\Administrator\Desktop\code1\codevolume.xlsx',excel);

disp('done!');

%{

In folder sGM are the smoothed gray matter imges of all subjects.

In folder totalGMvolume are the .mat files containing brain volume information of all subjects generated by Cat12 after segmentation.

rbasalganglia.nii is the mask of bilateral basal ganglia.

%}
